# Home Health Nurses’ Perceptions of Caring for Persons With Severe and Persistent Mental Illnesses

**DOI:** 10.1177/10783903241252165

**Published:** 2024-05-07

**Authors:** Kiernan Riley, Judith E. Hupcey

**Affiliations:** 1 Kiernan Riley, PhD, RN, Fitchburg State University, Fitchburg, MA, USA; 2 Judith E. Hupcey, EdD, CRNP, FAAN, Penn State Ross and Carol Nese College of Nursing, Hershey, PA, USA

**Keywords:** home healthcare, severe mental illnesses, home nursing

## Abstract

**BACKGROUND:**

Severe and persistent mental illnesses (SPMIs) affect a significant portion of the adult population in the United States. Despite their increased medical disease burden, individuals with SPMIs often lack access to appropriate medical care. Home health services offer cost-effective options for caring for this population in the comfort of their homes. However, little is known about the perceptions of home health nurses providing care to persons with SPMIs, and how they are adjusting care to persons with SPMIs

**AIMS:**

This study aimed to explore home health nurses’ perspectives on caring for persons with SPMIs.

**METHODS:**

Using a grounded theory approach, individual semi-structured interviews were conducted with home health and home hospice nurses. The research questions focused on the nurses’ experiences, barriers and facilitators to care, and the impact of the home environment on caring for persons with SPMIs. Data analysis followed coding procedures outlined in grounded theory, resulting in the development of an axial coding model.

**RESULTS/CONCLUSIONS:**

The findings provide valuable insights into the challenges and opportunities faced by home health nurses when providing care for individuals with SPMIs. The outcomes of this study are intended to contribute to the understanding of current care practices and can guide the allocation of resources to improve care for this vulnerable population, such as incorporating training specific to persons with severe psychiatric illnesses.

Severe and persistent mental illnesses (SPMIs) refer to serious psychiatric disorders characterized by the presence of a Diagnostic and Statistical Manual of Mental Disorders, Fifth Edition, (*DSM*-5) diagnosis, a significant level of patient dysfunction, and a diagnosis lasting >2 years (Zumstein & Riese, 2020). Persons with SPMIs comprise over 5% of the United States adult population (National Institute of Mental Health, 2023). These individuals often cannot afford effective medical care throughout their illness (Donald & Stajduhar, 2019), despite experiencing enhanced medical disease burden (De Hert et al., 2018). Home health services are cost-effective options to care delivery for persons with SPMIs in the home or community setting. They can provide an optimal approach to care for persons with difficulty leaving their homes for medical care (Lizano-Díez et al., 2022). However, researchers have not explored the provision of home health and the perceptions of home health nurses providing care to persons with SPMIs. Exploring nursing views to establish insights into current care practices is necessary before determining the resources needed to improve care for this population. Therefore, this study aims to fill this gap and explore home health nurse perspectives of caring for persons with SPMIs in a home setting. Home health and hospice nurses were chosen specifically due to their shared care-provision space, which is often the home setting. In these settings, the nurse is providing care alone, as opposed to with coworkers nearby in a team.

A grounded theory approach was utilized to collect, analyze, and report findings to understand better the home health nurses’ perception of caring for persons living with SPMI(s). The following research questions were used to guide the study:

1. How do nurses experience caring for persons with SPMIs at home?
2. What are the barriers and facilitators to home healthcare for persons with SPMIs?
3. How does the home environment impact care for persons with SPMIs?

## Methods

A grounded theory approach was used to explore the experiences of home health nurses providing care to the SPMI population. This allowed for the exploration of an emerging topic. It served as a foundational, flexible methodology to explore the process of providing care to persons with SPMIs. Individual, semi-structured interviews were conducted between the researcher and home health nurse participants for data collection.

Following institutional review board approval (IRB# 00014624), snowball and purposive sampling were utilized to recruit nurses. Flyers were posted in targeted social media groups specific to home health and hospice nurses practicing in the United States. Following a nurse’s response to the flyer, a Zoom interview was scheduled if the inclusion criteria were met and the nurse verbally consented. Zoom security was maintained through individual links, password-protected zoom rooms, and recordings removed from account following download to password-protected and encrypted computer. Inclusion criteria were English-speaking nurses (licensed practical nurse [LPN] or registered nurse [RN]) currently practicing in home health and/or home hospice or having practiced in home health/hospice within the previous year.

Interviews were conducted using the Zoom link provided, following informed consent discussion and verbal consent, so no identifiers were collected. Interviews were audio recorded only and transcribed verbatim by team member (KR). All potentially identifying information was removed from the final transcript. The semi-structured interviews were facilitated with an investigator-developed interview guide. Participants were provided with a definition of SPMIs, then asked follow-up questions. Example questions included:

1. Can you tell me about the SPMI population that you’ve cared for in a home health setting?
2. Can you tell me about your experiences of working with persons with SPMI in home health?

Following the interview, participants were offered a $10 Amazon gift card, at which time they could provide their email address. All audio and cleaned transcribed interviews were stored securely on the researcher’s password-protected laptop under the university’s password-protected storage system.

The research team coded data from transcripts using the grounded theory approach detailed by Corbin and Strauss (2008). This consists of a first stage of opening coding, a secondary stage of axial coding, and a final stage of selective coding. Coding procedures included using the definitions and tactics Corbin and Strauss (2008) delineated. Coding was done line-by-line, with ongoing iterative discussions between the researchers (KR, JH). The phenomenon of interest was nurses providing home healthcare to persons with SPMIs.

Following transcription, data were entered into NVivo software for analysis. A sequence of open, axial, and selective coding described by Corbin and Strauss (2008) was utilized to identify preliminary insights into the developing grounded theory. Axial coding categories included the causal factors, contexts, actions/interactions, intervening conditions, and consequences as delineated within this sector of grounded theory. Coding was an iterative process throughout these stages. Ongoing collaborative research team meetings were used throughout data coding and analysis to enhance credibility.

## Results

**Figure 1.**
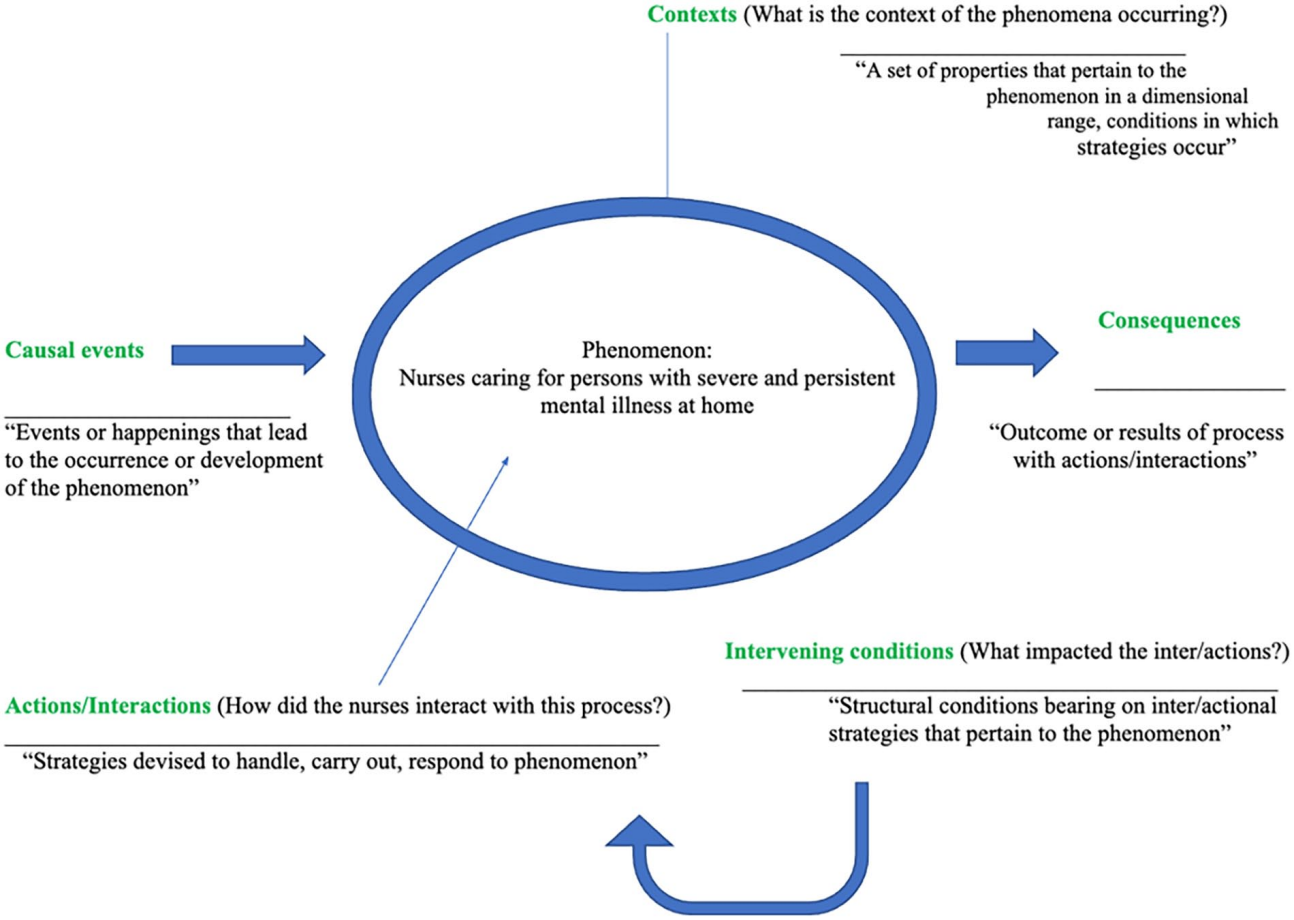
Adapted Axial Coding Framework. *Note*. The model was organized according to axial definitions provided by Corbin & Strauss, 2008.

The final data set consisted of 24 one-on-one interviews. The average length of interviews was approximately 25 minutes, translating to approximately seven pages of written text per interview. An exhaustive list of codes, categories, and axial coding was developed. All codes were directly from transcribed interviews, which were then collapsed into categories, and categories were then axial coded into the developed axial coding model. The model was organized according to axial definitions provided by Corbin and Strauss (2008). The model is presented in Figure 1. Each corresponding axial coding category will be discussed.

### Causal Events

Causal events were the reasons a person with an SPMI needed care from the home health/hospice nurse. Participants discussed the reasons persons with SPMIs receive home health/hospice services. These include primary diagnoses of psychiatric and physical origin, both of which were identified as appropriate for home health. One participant said:

So, you’d have whatever the medical issue diagnosis was that we were seeing them for, and then you’d also see whether they have a mental health history or have history of depression, or they’re on some psych meds too, or they’re very anxious. (T23)

## Contexts

Two broad categories of context for caring for persons with SPMIs emerged. First, “home,” which included the physical aspects of the home, patient, and family. Within this category, the physical characteristics of houses were discussed as both barriers and facilitators, as well as familial and patient considerations which may impact care. The second category, “nursing scope of practice,” also emerged as a context of care, which described the broad scope of nursing practice with various populations and the limitations of their skill sets.

### Home

In terms of the physical aspects of the home, participants discussed how the home could impact the patient’s stability either positively or negatively. Overall, participants agreed that the home is an appropriate place for care given a supportive home environment.

I think if you have a supportive home environment, whether or not the person has a severe and persistent mental illness, you’re more likely to reach the goals that are indicated for that patient. (T001)

Participants explicitly discussed barriers to care. These spanned the physical home, family, and patients. Participants explained that barriers included poor familial support, families with a mental illness, patient symptoms as barriers, stigmatization, and a depleted home environment.

Poor familial support was identified repeatedly as a major barrier to caring for persons with SPMIs at home. Participants identified the role of the family in typical home health/hospice care. However, they acknowledged that many persons with SPMIs have a strained or lacking relationship with family members, impeding their care potential at home. An example quote to support this belief is:

I would say really just a lack of support. So, it’s hard for the patients to continue doing well when they don’t have a support system in place (t025)

Participants also identified that when patients have family members around, they often suffer from similar mental illnesses. This creates a further barrier due to a lack of suitable caregivers and caregivers who often interfere with care. One participant stated:

I don’t know if the patient had psych issues, but the family had a lot of psych issues for sure. They had a lot of angst among the primary caregiver. That was definitely a dynamic that made me want to quit. (t006)

Patients’ actions because of their SPMI also were discussed as barriers. These actions included nonadherence to care or medications, visit cancelations, distrust over healthcare providers, and poor coping. Participants attributed each of these barriers to a psychiatric illness as “symptoms.” Nonadherence to medication regimens was discussed as a highly impactful barrier to effective care, and often not due to the fault of the patient but of their psychiatric illness. One participant stated:

The biggest problem that I see is them just not being compliant with their medications. (T025)

Other patient considerations impacting care included distrust over healthcare providers, patients not “fitting into” societal constraints, the need for routine, and stigmatization. Participants described that persons with SPMIs often do not trust the healthcare system, leading to difficulties with rapport building. Furthermore, participants stated that persons with SPMIs often “live outside the norm” and try to reconcile care for these patients while meeting their preferences for living. For example, one participant stated:

What we consider normal routine is really not normal for someone who’s lived a life on the outskirts of the norm. I’m trying to tell them they should be asleep at a certain time, and they shouldn’t be watching TV at a certain time and eating at a certain time. All of that is not necessarily their norm (T011)

However, although persons with SPMIs were identified as living outside the “norm,” the need for an established routine for the patient was discussed as a critical necessity. Deviation from routine was seen as a barrier to care. One participant stated:

It would be different in the sense that if you have someone with heart failure at the end of life, their mental status is a little different, but if you have someone with psychiatric issues, you still need to maintain a certain routine. I have found that if you get out of their ordinary routine, versus someone who’s just medical like heart failure, it can really alter their comfort, their anxiety, their pain, everything about that (T003).

Participants also identified the impact of stigmatization on the SPMI population. Stigma was described as coming from society and those around persons with SPMIs and from inside the healthcare system, all of which have detrimental outcomes for persons with SPMIs. One participant stated:

I know that I’ve had nurses that are very reluctant to go just because of the stigma of the psychiatric diagnosis. So, I know that has a negative connotation to some of the nurses, just because I guess of the unknown (T20)

### Nursing Scope of Practice

Nursing scope of practice emerged as a category that helped describe the context of care provision to the SPMI population in the home. Participants described both barriers and facilitators of the nursing scope of practice. For instance, participants stated that all nurses are trained in mental health and, therefore, should be capable of handling the SPMI population effectively in any context. One participant said:

I’m thinking to myself; MOST NURSES should be able to competently handle depressed, even very depressed, patients and then patients with anxiety. They should be able to intervene, even if that meant making a mental health referral (T013)

Despite the universality of nursing practice, nursing scope limitations were also discussed. Nurses often felt they were practicing outside their scope, particularly trying to fill the shoes of licensed counselors or social workers. This resulted in significant frustration and feelings of futility for nurses. One participant stated:

We wear many hats to the best of our ability. I mean, I’m not even going to brag and say that I’m good at all these things, I try, but I wasn’t formally trained, of course. (T017)

## Actions/Interactions

Actions/interactions are the processes nurses take when caring for persons with SPMIs and how they provide care. Two major categories emerged: nurse tactics and the role of the nurse. Nurse tactics refer to how the nurse provides care and the decisions the nurse makes when providing care to persons with SPMIs at home, while the role of the nurse refers to tasks described as necessary when providing care.

### Nurse Tactics

Nurse tactics included actions the nurse took, such as increasing visit frequency when needed, maintaining a strict schedule, keeping a consistent staff assignment, and remaining firm with the patient. These tactics were identified as having a heightened priority within the SPMI population as each helps build and maintain nurse-patient rapport. Rapport was identified as crucial in caring for the SPMI population, particularly in their home setting. Rapport was necessary not only for patients but for nurses as well. Participants described needing to become comfortable and trusting the patient before being able to “relax” in the home setting. Participants stated they could accomplish more with the patient by building and maintaining trust. One participant stated:

I think going in and being able to build that rapport with the patient is the most important thing. It’s for them seeing that they can trust you. (T025).

The importance of communication and listening also was stressed. Communication skills with patients and interdisciplinary team members were identified as necessary components of care. Listening, particularly to patients and their needs, was highlighted, even if not a medical intervention.

. . . ultimately, the difference they’re going to remember is if you listened. Sometimes you’re just their company, because they have no family, or don’t speak to them, nobody who visits them just calls them on the phone. You’re making a really big difference in their life just by coming in and listening to them. . .(T006)

### Role of Nurse

Within the nurse’s role, the codes included differentiating psychiatric symptoms and managing long-term psychiatric medication usage. Participants also discussed the difficulty in caring for persons with SPMIs due to poor medical history/documentation, and at times, there was no noted psychiatric history, despite a severe psychiatric illness. “Hidden” or undiagnosed psychiatric symptoms impeded care, leaving the nurse unprepared. Often, nurses felt as if they were “guessing” that there was a psychiatric illness instead of walking in prepared with a diagnosis. Furthermore, participants highlighted that, at times, a medication list is the only indication of a psychiatric problem.

But we do go into many homes that are very unsavory if that’s the right word, and I think in the back of my mind, it always registers. “There is something going on mentally with these people; why would you live like this?” To me, there has to be some sort of risk behaviors, all these risk behaviors. In some way, shape, or form, psychiatric issues. Something there. (T005)

## Intervening Conditions

Intervening conditions consist of structural aspects of healthcare systems that impact the nurse’s strategies (actions/interactions) when caring for the SPMI population in a home setting. Three main categories emerged: agency resources, other resources, and nurse preparation.

### Agency Resources

Agency resources and other resources are referred to as facilitative resources within the home health/hospice agency and the community. Agency resources included knowledgeable and available supervisors, case conferences, interdisciplinary teams, and set agency guidelines for care. The most helpful agency resources were the presence of a designated psychiatric nurse. Participants described the collaborations with agency psychiatric nurses as necessary and fruitful. One participant stated:

. . . we do have access to behavioral health. I do believe that some companies, companies are built different, and I do believe, currently, I am in a better place than I was before because we have access to behavioral health nurses (T017).

Within agency resources, social workers were identified as one of the most influential and necessary team members. The impact of social workers could not be understated and was mentioned by nearly all participants. Some examples include:

We do also have an excellent backup of our MSWs, our social worker, where if you feel anything is not quite right, you have to contact our social worker, and she gets involved in the case immediately (T005)

### Other Resources

Other resources included community resources such as Area Agencies on Aging, Adult Protective Services, transportation services, local crisis numbers, free healthcare clinics, and local psychiatric hospitals or institutions. These were identified as crucial in coordinating effective care for the SPMI population. However, many participants stated that the resources, albeit essential, are difficult to access. One participant stated,

There’s so much more to it and the resources while there are lots of—I have a whole binder filled with resources. It’s not that easy to access. Everybody says, ‘Oh, use the resources while they’re there.’ But again, you’re jumping through a ton of hoops (T017)

### Nurse Preparation

Nurse preparation was a category providing insights into how nurses’ educational, professional, and personal experiences impacted their care of persons with SPMIs. Furthermore, participants identified limitations to their own preparation and knowledge when caring for persons with SPMIs.

Previous professional experiences were described as the most important factor in preparation for caring for the SPMI population, along with self-education. Nurses stated that their previous experiences as nurses or unlicensed personnel were significant in feeling more comfortable and impacted their choices when caring for persons with SPMIs at home. One participant stated,

I also worked at a teaching service for a hospital, and we had a lot of patients who had a lot of hallucinations. Maybe that’s why I feel that I’m ok. (T008)

Educational experiences were identified as necessary, but undergraduate education was noted to be significantly lacking in preparing nurses to care for psychiatric patients. Furthermore, participants identified that if you did not specialize in psychiatric nursing, you missed valuable skills. One participant indicated that stigmatization might impact the decision to specialize.

I think partly because we don’t specialize in it and partly because it’s so stigmatized, a lot of nurses, myself included, just don’t have that exposure and don’t have the training for it.

The preparation of the nurse, as well as the availability of agency and other community resources, were seen as impacting the actions/interactions of the nurse when entering the process of caring for persons with SPMIs in the home setting.

## Consequences

The axial coding category of consequences was interpreted as the outcomes identified by the participants. Within this, a category of nurse perceptions emerged, which consisted of nursing outcomes of caring for persons with SPMIs at home.

Within nurse perceptions, participants discussed nursing outcomes such as patients scaring nurses and threats to nurse safety, which often resulted in nursing danger anticipation. Participants identified an enhanced sense of alertness when caring for persons with SPMIs at home. This was due to both the patient population and the home setting. A participant stated,

You have to remain alert. A different kind of alert in the back of your mind that just because it’s going swimmingly in two seconds, they can lash out. They may not recognize you, and they think their world has changed in the blink of an eye. So, you remain on guard. A little more than usual. (T011)

Participants also discussed the frequent presence of weapons in homes, which enhanced feelings of danger. Guns, axes, and knives were each mentioned as inhibiting safety in homes and therefore impacted the care of persons with SPMIs. Nurses identified that when they sensed danger, care was often curtailed.

Oh yes, in that when nurses are afraid for their safety, if they think they’re in an unsafe environment—they are in and out of there in a flash. (T013).

Apprehension was noted as being present not just when caring for persons with SPMIs at home but for any home health case due to the unknowns associated with home health.

Even though I’ve done this for years, and I’ve got a lot of experience nursing behind me, and I’ve been in the field, there still is that little level of apprehension as the nurse going to that door for the first time, there really is. (T05).

Consequences for approaching care were also discussed. Participants described how they approached care differently with the SPMI population due to past experiences. Three codes emerged describing these considerations, including: “Doing the best we can,” “Meet them where they’re at,” and “Hands are tied.” Given the difficulties, nurses said they were doing their best with this population. Example quotes include:

I think in a lot of ways, we’re going about it the same way in trying to do the best you can without going into it with a bias or with a prejudice and trying to do what’s right for them. (T024)

I mean, as far as my agency goes and the doctors, I mean, I feel like we’re all doing the best that we can with this population in a home setting (T025).

This included meeting patients “where they’re at” to provide the best care. One nurse stated:

I think there’s a huge comfort in nurses being able to meet patients where they’re at and in their home and in the comfort of their home and then the as clinician going in being able to see them. (T016).

Other consequences included prioritization of non-psychiatric symptoms and patients. Nurses described patients with SPMIs as being on the “back burner” or “pushed to the wayside.” One participant described psychiatric symptoms as: “It’s more like this is something else that you’re dealing with rather than our primary focus” (T024). Even more pointedly, one participant described psychiatric symptoms as a roadblock in caring for persons with SPMIs.

When I get there to try to provide the medical care, the mental health becomes an automatic roadblock. Because they just simply cannot get on board with the plan of care the same way that they would if they didn’t have that issue (T017).

## Discussion

The home setting can be an appropriate place to receive care for persons with SPMIs if the nurses are given proper training, preparation, and resources. However, the current nursing experience in providing care to persons with SPMIs in the home sector is varied and often extremely challenging, if it occurs at all. Furthermore, the home must be conducive to effective care and support, including family and/or caregivers, and a clean, peaceful environment. However, this is not always the case for persons with SPMIs.

Throughout the interviews, nurses described their experiences of caring for persons with SPMIs as generally limited. When they did care for persons in this population, it was often with apprehension due to poor preparation. Nurses who felt comfortable with SPMI assignments stated it was due to previous professional experiences caring for persons with SPMIs in a different venue in the past. Facilitators enhancing the nurse experience included agency support, on-the-job education and training, and having other staff available to support and backup when necessary.

Throughout the axial coding model, barriers and facilitators were noted by nurse participants. Barriers were identified within nurses, healthcare systems, patients, patient lifestyles, and family systems. Nurses identified their lack of training or comfort with psychiatric patients. A quantitative study by Joubert and Bhagwan (2018) found that psychiatric nurses reported frustration with illness denial in the mental health population. Furthermore, enhanced feelings of frustration and anger, as well as enhanced exposure to violence and abuse, were noted among psychiatric nurses. Similar sentiments emerged in this study for home health nurses working with mental health patients.

Throughout this study, nurses also identified aspects of the home that were barriers or facilitators to care. Barriers noted within the literature for home-bound patients, in general, can include clutter, dirty homes, limited supplies (Adams et al., 2021), small rooms/bathrooms, and poorly portioned homes (Pettersson et al., 2021). Nurses identified significant barriers for persons with severe mental illnesses, who have fewer accessible housing choices, as well as infestation issues, issues with housemates, and violent homes, including firearms and other weapons. System-wide barriers, including fear of persons with SPMIs interacting with healthcare, and siloing of healthcare services are also noted within the literature but were not robustly discussed by participants (McNamara et al., 2018). Within the interviews, participants felt poor home conditions were more often encountered with persons with SPMIs or suspected SPMIs among caregivers. However, important facilitators were also noted, including patient comfort being enhanced in the home space and seeing the holistic health view of the patient when allowed into their home. Therefore, the home environment can be an excellent option for care provision; however, persons with SPMIs may need more support to enhance this environment. As it has been shown, the home environment can be beneficial to patient health outcomes and increase the likelihood of terminal patients dying at home (which is often the goal; Shepperd et al., 2021). Efforts to enhance the ability of persons with SPMI to choose to remain at home for effective care should be prioritized.

While caring for persons with SPMIs at home can be challenging, the nurses did identify tactics within the nursing scope of practice that can enhance their nursing care experience, which included focusing on rapport, keeping consistent staff, maintaining firm boundaries with the patient, and keeping a tight schedule, with more support visits as needed. Boundaries, specifically, become highlighted in the home setting and are frequently discussed in the literature as a difficult but necessary aspect of home care to navigate (Abrahms et al., 2019); however, flexibility and adaptation are necessary. Other tactics nurses noted in the present study were reinforced as effective tactics, particularly prioritizing rapport and boundaries in the home (Hill, 2005; Terpstra & Williamson, 2019).

It should also be noted that while it did not emerge as a label by participants, stigmatization among healthcare and society toward persons with SPMIs contributes to lower quality of life in patients (Relyea et al., 2019). Stigmatization also was noted in some of the interviews. For example, nurses used phrases such as “the crazies” or “those psychos” when referring to this group. It is necessary for stigmatization to be evaluated and for nurses to question their own personal and professional stigmas that they may be imprinting on their patient populations. Implicit bias and stigmatization from healthcare workers can be detrimental to clinical judgment and subsequent behavior toward patients with SPMIs (Fitzgerald & Hurst, 2017). Factors that may contribute to nurse attitudes toward persons with SPMIs include length of experience, individual empathy capabilities, and the type of presenting illness, as there tends to be more bias toward illnesses such as substance abuse than toward schizophrenia (Hsiao et al., 2015).

## Implications

Results from this study provide insights into the needs of nurses caring for persons with SPMIs at home. It is clear from interviews that expanded community and agency resources are necessary to best assist nurses in providing appropriate care to the SPMI population. Furthermore, enhanced training or education is essential for nurses without experience with this population, and ongoing education can potentially enhance both the nurses’ and patients’ experiences. As home care options expand for patients and those with SPMIs, it is imperative for nurses to understand how to properly care for them.

## Limitations

While this study provides valuable insights into the experiences and perspectives of individuals with SPMIs, it is important to acknowledge certain limitations inherent in the research design. One notable limitation is the reliance on symptoms as indicators of SPMIs by the nurses. The study utilized, in some cases, assessment of symptoms as a basis for understanding and categorizing participants’ mental health conditions, which may introduce subjectivity and potential inaccuracies. The use of symptom-based criteria may not fully capture the complexity and variability of SPMIs, as these conditions often manifest uniquely in each individual. However, since this study is examining the nursing process of caring for persons with SPMIs, it was important to include nurse interpretations of this concept, as their perception of this will subsequently impact care. Further research is needed relating to nurse perceptions of mental illnesses in relation to SPMI. Furthermore, demographic data were not collected on participants beyond inclusion criteria. This information could strengthen understanding the knowledge base of the results.

Researcher bias is always a concern in qualitative research, particularly in this case when the primary investigator is a home health/hospice nurse; however, the axial coding and sufficient bracketing helped address researcher bias.

## Conclusions

This qualitative grounded theory study explored the experiences of home health and hospice nurses caring for persons with SPMIs at home. Nurses identified that while the home is an appropriate place to receive care, there are necessary resources and preparation which should be addressed. There is a need for growing research with this population and in this care venue, with considerations for the context in which persons are presenting with their illness and receiving care.
